# Hypoperfusion intensity ratio is associated with follow-up infarct volume in medium vessel occlusions: A multicenter multinational study

**DOI:** 10.1016/j.neurot.2025.e00713

**Published:** 2025-08-18

**Authors:** Vivek Yedavalli, Hamza Adel Salim, Dhairya Lakhani, Basel Musmar, Nimer Adeeb, Davide Simonato, Yan-Lin Li, Orabi Hajjeh, Muhammed Amir Essibayi, Nils Henninger, Sri Hari Sundararajan, Anna Luisa Kühn, Jane Khalife, Sherief Ghozy, Luca Scarcia, Leonard LL. Yeo, Benjamin YQ. Tan, Robert W. Regenhardt, Jeremy J. Heit, Aymeric Rouchaud, Jens Fiehler, Sunil Sheth, Ajit S. Puri, Christian Dyzmann, Marco Colasurdo, Leonardo Renieri, João Pedro Filipe, Pablo Harker, Răzvan Alexandru Radu, Mohamad Abdalkader, Piers Klein, Takahiro Ota, Ashkan Mowla, Kareem El Naamani, Pascal Jabbour, Arundhati Biswas, Frédéric Clarençon, James E. Siegler, Thanh N. Nguyen, Ricardo Varela, Amanda Baker, David Altschul, Nestor R. Gonzalez, Markus A. Möhlenbruch, Vincent Costalat, Benjamin Gory, Christian Paul Stracke, Constantin Hecker, Gaultier Marnat, Hamza Shaikh, Christoph J. Griessenauer, David S. Liebeskind, Alessandro Pedicelli, Andrea M. Alexandre, Tobias D. Faizy, Illario Tancredi, Erwah Kalsoum, Boris Lubicz, Aman B. Patel, Maurizio Fuschi, Max Wintermark, Adrien Guenego, Adam A. Dmytriw, MAD MT Investigators, Abdelaziz Amllay, Achala Vagal, Adrien ter Schiphorst, Ajith J. Thomas, Anil Gopinathan, Anne Dusart, Carolina Capirossi, Charbel Mounayer, Charlotte Weyland, Cheng-Yang Hsieh, Christopher J. Stapleton, Erwah Kalsoum, Flavio Bellante, Géraud Forestier, Hamza Shaikh, Hugo H. Cuellar-Saenz, Iacopo Valente, Igor Sibon, James D. Rabinov, Jérôme Berge, Jessica Jesser, Juan Carlos Martinez-Gutierrez, Kevin Premat, Lina Chervak, Lukas Meyer, Mahmoud Elhorany, Miguel Quintero-Consuegra, Mohammad Ali Aziz-Sultan, Monika Killer-Oberpfalzer, Peter T. Kan, Priyank Khandelwal, Ramanathan Kadirvel, Robert Fahed, Sergio Salazar-Marioni, Shogo Dofuku, Simona Nedelcu, Stavropoula I. Tjoumakaris, Suzana Saleme, Xavier Barreau, Yasmin Aziz

**Affiliations:** at Department of Neurosurgery, Thomas Jefferson University, Philadelphia, PA, USA; au Department of Neurology and Radiology, University of Cincinnati, USA; av Department of Neurology, Gui de Chauliac Hospital, Montpellier University Medical Center, France; aw Departments of Neurological Surgery & Radiology, Mayo Clinic, Rochester, MN, USA; ax Department of Medicine, Yong Loo Lin School of Medicine, National University of Singapore, Singapore; ay Department of Neurology, Hôpital Civil Marie Curie, Charleroi, Belgium; az Interventistica Neurovascolare, Ospedale Careggi di Firenze, Florence, Italy; ba University Hospital of Limoges, Neuroradiology Department, Dupuytren, Université de Limoges, XLIM CNRS, UMR 7252, France; bb Sektion Vaskuläre und Interventionelle Neuroradiologie, Universitätsklinikum Heidelberg, Heidelberg, Germany; bc Neurology Department, Sin-Lau Hospital, Tainan, Taiwan, ROC; bd Department of Neurosurgery and Interventional Neuroradiology, Louisiana State University, LA, USA; be Department of Neuroradiology, Henri Mondor Hospital, Creteil, France; bf Cooper Neurological Institute, Cooper University Hospital, Cooper Medical School of Rowen University, Camden, NJ, USA; bg UOSA Neuroradiologia Interventistica, Fondazione Policlinico Universitario A. Gemelli IRCCS Roma, Italy; bh Neurology Department, Bordeaux University Hospital, Bordeaux, France; bi Interventional Neuroradiology Department, Bordeaux University Hospital, Bordeaux, France; bj Department of Neurology, UTHealth McGovern Medical School, Houston, TX, USA; bk Department of Neuroradiology, Pitié-Salpêtrière Hospital, Paris, France; bl GRC BioFast, Sorbonne University, Paris VI, USA; bm Department of Diagnostic and Interventional Neuroradiology, University Medical Center Hamburg-Eppendorf, Hamburg, Germany; bn Department of Neurosurgery, Cedars-Sinai Medical Center, Los Angeles, USA; bo Department of Neurosurgery, Brigham and Women's Hospital, Harvard Medical School, Boston MA, USA; bp Departments of Neurology & Neurosurgery, Christian Doppler Clinic, Paracelsus Medical University Salzburg, Austria; bq Department of Neurosurgery, University of Texas Medical Branch, Galveston, TX, USA; br Department of Endovascular Neurosurgery and Neuroradiology NJMS, Newark, NJ, USA; bs Department of Medicine, Division of Neurology, The Ottawa Hospital, Ottawa Hospital Research Institute and University of Ottawa, Ottawa, Ontario, Canada; bt Department of Neurosurgery, Tokyo Metropolitan Tama Medical Center, Tokyo, Japan; bu Department of Neurology, University of Massachusetts Chan Medical School, Worcester, MA, USA; bv Department of Neurology, University of Cincinnati Medical Center, Cincinnati, OH, USA; a Department of Radiology, Division of Neuroradiology, Johns Hopkins Medical Center, Baltimore, MD, USA; b Department of Neuroradiology, MD Anderson Medical Center, Houston, TX 77030, USA; c Department of Neuroradiology, Rockefeller Neuroscience Institute, West Virginia University, Morgantown, WV 26505, USA; d Department of Neurosurgery, Thomas Jefferson University, Philadelphia, PA, USA; e Department of Neurosurgery and Interventional Neuroradiology, Louisiana State University, LA, USA; f Department of Neurology, University of Massachusetts Chan Medical School, Worcester, MA, USA; g Department of Neuroradiology, Oxford University Hospitals NHS Foundation Trust, Nuffield Departments of Clinical Neurosciences and Surgical Sciences, University of Oxford, UK; h Department of Neurological Surgery and Montefiore-Einstein Cerebrovascular Research Lab, Montefiore Medical Center, Albert Einstein College of Medicine, Bronx, NY, USA; i Department of Endovascular Neurosurgery and Neuroradiology NJMS, Newark, NJ, USA; j Division of Neurointerventional Radiology, Department of Radiology, University of Massachusetts Medical Center, Worcester, MA, USA; k Cooper Neurological Institute, Cooper University Hospital, Cooper Medical School of Rowen University, Camden, NJ, USA; l Nuffield Department of Primary Care Health Sciences and Department for Continuing Education (EBHC Program), University of Oxford, Oxford, UK; m Department of Neuroradiology, Henri Mondor Hospital, Creteil, France; n Department of Medicine, Yong Loo Lin School of Medicine, National University of Singapore, Singapore; o Division of Neurology, Department of Medicine, National University Hospital, Singapore; p Neuroendovascular Program, Massachusetts General Hospital & Brigham and Women's Hospital, Harvard Medical School, Boston, MA, USA; q Department of Interventional Neuroradiology, Stanford Medical Center, Palo Alto, CA, USA; r University Hospital of Limoges, Neuroradiology Department, Dupuytren, Université de Limoges, XLIM CNRS, UMR 7252, France; s Department of Diagnostic and Interventional Neuroradiology, University Medical Center Hamburg-Eppendorf, Hamburg, Germany; t Department of Neurology, UTHealth McGovern Medical School, Houston, TX, USA; u Neuroradiology Department, Sana Kliniken, Lübeck GmbH, Lübeck, Germany; v Department of Interventional Radiology, Oregon Health and Science University, Portland, OR 97239, USA; w Interventistica Neurovascolare, Ospedale Careggi di Firenze, Florence, Italy; x Department of Diagnostic and Interventional Neuroradiology, Centro Hospitalar Universitário Do Porto, Porto, Portugal; y Department of Neurology, University of Cincinnati Medical Center, Cincinnati, OH, USA; z Department of Neuroradiology, Gui de Chauliac Hospital, Montpellier University Medical Center, France; aa Departments of Radiology & Neurology, Boston Medical Center, Boston, MA, USA; ab Department of Neurosurgery, Tokyo Metropolitan Tama Medical Center, Tokyo, Japan; ac Division of Stroke and Endovascular Neurosurgery, Department of Neurological Surgery, Keck School of Medicine, University of Southern California (USC), 1200 North State St, Suite 3300, Los Angeles, CA, USA; ad Department of Neurosurgery, Westchester Medical Center at New York Medical College, Valhalla, NY, USA; ae Department of Neuroradiology, Pitié-Salpêtrière Hospital, Paris, France; af GRC BioFast, Sorbonne University, Paris VI, USA; ag Department of Neurology, Centro Hospitalar Universitário Do Porto, Porto, Portugal; ah Department of Neurosurgery, Cedars-Sinai Medical Center, Los Angeles, USA; ai Sektion Vaskuläre und Interventionelle Neuroradiologie, Universitätsklinikum Heidelberg, Heidelberg, Germany; aj Department of Interventional Neuroradiology, Nancy University Hospital, Nancy, France; ak INSERM U1254, IADI, Université de Lorraine, 54511 Vandoeuvre-les-Nancy, France; al Department of Radiology, Interventional Neuroradiology Section, University Medical Center Münster, Germany; am Departments of Neurology & Neurosurgery, Christian Doppler Clinic, Paracelsus Medical University Salzburg, Austria; an Interventional Neuroradiology Department, Bordeaux University Hospital, Bordeaux, France; ao UCLA Stroke Center and Department of Neurology Department, UCLA, Los Angeles, CA, USA; ap UOSA Neuroradiologia Interventistica, Fondazione Policlinico Universitario A. Gemelli IRCCS Roma, Italy; aq Department of Radiology, Neuroendovascular Program, University Medical Center Münster, Germany; ar Department of Neurology, Hôpital Civil Marie Curie, Charleroi, Belgium; as Department of Diagnostic and Interventional Neuroradiology, Erasme University Hospital, Brussels, Belgium

**Keywords:** Medium Vessel Occlusion (MeVO), Hypoperfusion Intensity Ratio (HIR), Follow-Up Infarct Volume (FIV), Mechanical Thrombectomy, Collateral Circulation, CT Perfusion (CTP), Tmax Thresholds, Acute Ischemic Stroke, Distal Occlusions, Stroke Ou

## Abstract

Medium vessel occlusion (MeVO) contributes significantly to acute ischemic stroke (AIS). The hypoperfusion intensity ratio (HIR), reflecting collateral circulation via the ratio of Tmax >10s to Tmax >6s volumes, predicts infarct progression in large-vessel occlusions but is unstudied in MeVOs. In this multicenter, multinational retrospective study, we evaluated consecutive patients with MeVO who underwent mechanical thrombectomy with or without intravenous thrombolysis. Inclusion required available follow-up imaging and pretreatment CT perfusion. Follow-up infarct volume (FIV) was measured on CT or MRI 12–36 ​h post-procedure. Univariable and multivariable linear regression models were used to identify predictors of FIV, with HIR as the primary variable of interest. Among 147 patients (median age 75 years, 57 ​% female), univariable analysis showed HIR was significantly associated with larger FIV (β ​= ​80 ​mL; p ​< ​0.001). After adjusting for confounders, HIR remained independently associated with FIV (β ​= ​40 ​mL; p ​< ​0.001). Tmax >10 ​s showed the strongest correlation with FIV (r ​= ​0.56; p ​< ​0.001). These findings suggest that higher HIR correlates with larger infarct volumes, underscoring the prognostic role of collateral failure in MeVO and highlighting HIR as a potential imaging marker to guide treatment and outcome prediction.

## Introduction

Medium vessel occlusion (MeVO) involves the M2-M4 segments of the middle cerebral artery (MCA), segments of the anterior cerebral artery (ACA), and vertebrobasilar branches. MeVO stroke is a significant subset of acute ischemic stroke (AIS), accounting for approximately 25–40 ​% of cases. While the occluded vessels are often smaller compared to large vessel occlusions (LVO), they are of particular clinical importance due to their potential to cause disabling symptoms [1,2].

Advanced neuroimaging allows for more precise measurement of follow-up infarct volume (FIV), a crucial parameter in the evaluation of stroke outcomes [[3], [4], [5]]. The extent of FIV is intricately correlated with the extent of neurological impairments and the overall functional outcome [[6], [7], [8]]. Recent studies highlight its prognostic value, with specific thresholds such as ≤15 ​mL predicting favorable outcomes and ≥40 ​mL associated with poor prognosis [9]. While FIV provides a direct measure of ischemic damage, its interplay with pretreatment imaging parameters remains underexplored in MeVOs.

Collaterals are a known imaging parameter of infarct growth rate [10,11] in AIS patients. CT and MR perfusion imaging, as an aspect of the pretreatment stroke workup, provides valuable information on collateral status (CS) [12,13]. These collaterals represent the secondary network of vessels that maintain blood flow to the brain tissue in the presence of an occlusion in the primary vessels [[14], [15], [16], [17]]. Moreover, the presence of robust collateral circulation is frequently associated with improved outcomes in AIS [14,18].

The hypoperfusion intensity ratio (HIR) is defined as the time to maximum (Tmax) ​> ​10 ​s volume divided by the Tmax >6 ​s volume [19]. HIR is a well-established quantified perfusion imaging parameter that serves as a surrogate for tissue level collaterals in AIS-LVO patients [10,11,[20], [21], [22], [23]] with a higher HIR indicating worse collaterals.

In this study, we aim to investigate the parameters associated with FIV in patients with MeVO stroke. We hypothesize that the higher HIR, which represents poor compensatory response through collateral routes, is associated with higher FIV on FLAIR imaging.

## Methods

This investigation is part of an analysis of the Multicenter Analysis of primary Distal medium vessel occlusions: effect of Mechanical Thrombectomy (MAD-MT) registry [16,17,[24], [25], [26], [27], [28], [29], [30], [31], [32], [33], [34], [35], [36], [37], [38], [39], [40], [41]]. The study received approval from the institutional review board or local ethical standards committee at each participating site, and informed consent from patients was waived given minimal patient risk. The de-identified data supporting this study's findings are available from the corresponding author upon reasonable request. This study is reported according to the Strengthening the Reporting of Observational Studies in Epidemiology (STROBE) guideline [42,43].

### Study population and setting

Inclusion criteria for this analysis were as follows: 1) Anterior circulation MeVO, as defined by Saver et al., encompassing occlusions in the M2 and M3 segments of the middle cerebral artery (MCA) or in the A2 segment of the anterior cerebral artery (ACA) [2]; 2) administration of mechanical thrombectomy (MT) with or without intravenous thrombolysis (IVT); 3) availability of presentation CT perfusion and follow-up FIV and data.

### Data collection and outcomes

Data were collected between September 2017 and July 2023. Data for this study were collected prospectively and reviewed retrospectively. The local neurointerventionalist reviewed all cases before sending their data to the MAD-MT consortium. They determined the angiographic treatment success before the data was sent to the consortium, which was self-reported by each center.

Baseline clinical and demographic characteristics were recorded for patients and included sex (male or female), age, hypertension, hypercholesterolemia, diabetes mellitus, atrial fibrillation, and smoking status. Pre-stroke modified Rankin Scale (mRS) score and occluded vessel were recorded. National Institutes of Health Stroke Scale (NIHSS) score was recorded at presentation. Baseline Alberta Stroke Program Early CT Score (ASPECTS) was assessed using non contrast head CT [44].

Other details of interest included antiplatelet and anticoagulation medication status, mothership versus drip-and-ship presentation [45], time from onset to puncture and recanalization, vital sign readings (blood pressure, heart rate), glycemic readings, anesthesia type (general, sedation, or local), access site (femoral or radial), and imaging data.

### Procedural and technical details

Treatment consisted of MT alone or MT ​+ ​IVT. MT access site, either femoral or radial artery, and endovascular strategy (aspiration, stent retriever, combined or rescue techniques) were left to the individual operator's discretion. Similarly, the number of passes was left to the treating physician's discretion and institutional guidelines. The final mTICI scores were site adjudicated.

### Imaging data

The CTP imaging was conducted using a pre-specific protocol. The CTP images were processed to produce Tmax maps, which were then utilized to calculate HIR. The Tmax thresholds of >6 ​s and >10 ​s were selected based on prior literature, where they have been validated as markers of collateral status and infarct risk in LVO patients [10,[20], [23]].

FIV was assessed on follow-up NCCT or MRI. If multiple follow-up scans were available, MRI was the preferred modality with a range of 12 ​h–36 ​h post MT. FIVs were calculated using either manual or semi-automated segmentation techniques and were reported per each center protocol. FIVs were calculated in milliliters (mL) by multiplying the number of voxels of the segmented ischemic lesions with its voxel size.

NCCT scans in our study were conducted using a helical scanning technique. The scans were performed with each slice having a thickness of 5 ​mm and a reconstruction resolution of 0.75 ​mm. The kilovoltage peak (kVp) was set at 120, and the milliampere-seconds (mAs) were set at 365. The rotation time of the CT scanner was maintained at 1 ​s, and the total acquisition time for each scan ranged between 6 and 8 ​s. The collimation of the scans was 128 ​× ​0.6 ​mm, and a pitch value of 0.55 was used. All scans were performed in a craniocaudal direction.

FLAIR imaging was conducted using Siemens Aera or Skyra scanners (Erlangen, Germany). The FLAIR sequence parameters on 3T were: Repetition Time (TR) was set in the range of 9000 ​ms, Echo Time (TE) around 105 ​ms, and Inversion Time (TI) at 2500 ​ms. Flip Angle: 160; Field of View (FOV): 42.8 ​× ​23 ​cm. The imaging was performed using either a 1.5 ​T or 3 ​T scanner. The slice thickness was maintained at 4 ​mm. At 1.5 ​T: Repetition Time (TR) was 7500 ​ms, Echo Time (TE) 78 ​ms, and Inversion Time (TI) at 2300 ​ms. Flip Angle: 180; FOV: 40.9 ​× ​22 ​cm. The slice thickness was maintained at 5 ​mm [17].

The FLAIR images were reviewed and analyzed by experienced neuroradiologists, focusing on the presence, location, and volume of ischemic lesions. The volume of ischemic lesions on FLAIR was determined by measuring the hyperintense regions indicative of ischemic tissue.

These imaging protocols varied among centers; however, the parameters provided are representative of those used in the majority of centers.

#### Statistical analysis

Continuous variables were described as medians with interquartile ranges (IQRs), while categorical variables were presented as frequencies and percentages. Our primary analysis involved the use of univariable linear regression to explore the association with FIV. This was followed by multivariable linear regression, including age, sex, and other variables with a p-value <0.1 in the univariate analysis. Effect estimates were presented as β coefficients with 95 ​% confidence intervals (CIs). To assess potential effect modification by occlusion site, we performed a subgroup analysis stratifying patients into M2 versus M3/A2 occlusions. An interaction term between HIR and occlusion subgroup was included in the regression model to evaluate whether the relationship between HIR and infarct volume differed by vascular territory.

Pearson's correlation coefficients (r) with 95 ​% CIs were calculated to assess the strength of associations between Tmax volumes (Tmax >10 ​s, >8 ​s, >6 ​s, and >4 ​s) and FIV.

Statistical significance was defined as *P* ​< ​0.05. R statistical software (version 4.3.0, R Project for Statistical Computing) and Rstudio statistical software (version 2023.03.0 ​+ ​386, Rstudio) were used for statistical analyses and data visualization.

## Results

### Patient demographics and clinical characteristics

The study included 147 patients (median age of 75 years [IQR, 66–84 years]; 63 males; 84 females). The initial occlusion site was predominantly in the M2 segment (88 ​%), with M3 and A2 segments accounting for 9.5 ​% and 2.0 ​% of cases, respectively. Pre-stroke mRS scores were 0–1 in 74 patients (86 ​%) and 0–2 in 82 patients (95 ​%). The ASPECTS was 9.00 (IQR, 8.00–10.00), and the baseline NIHSS score had a median of 11.0 (IQR, 7.0–16.0). The HIR had a median value of 0.32 (IQR, 0.20–0.57). Further details on baseline characteristics are provided in Table 1.

**Table 1:** Baseline patient demographics and clinical characteristics.

Variable^a^	N ​= ​147
Male, n (%)	63 (43)
Age, median (IQR)	75 (66, 84)
Hypercholesterolemia, n (%)	49 (33)
Hypertension, n (%)	115 (78)
Site of initial occlusion, n (%)	
*A2*	3 (2.0)
*M2*	130 (88)
*M3*	14 (9.5)
Diabetes, n (%)	26 (18)
Atrial fibrillation, n (%)	30 (20)
Current smokers, n (%)	30 (21)
Previous use of antiplatelet drugs, n (%)	50 (34)
Previous use of anticoagulant drugs, n (%)	20 (23)
Pre-stroke mRS (0–1), n (%)	74 (86)
Pre-stroke mRS (0–2), n (%)	82 (95)
ASPECTS, median (IQR)	9.00 (8.00, 10.00)
Baseline NIHSS, median (IQR)	11.0 (7.0, 16.0)
Hypoperfusion intensity ratio (HIR), median (IQR)	0.32 (0.20, 0.57)

a Abbreviations: mRS ​= ​Modified Rankin Scale, NIHSS ​= ​National Institutes of Health Stroke Scale, ASPECTS ​= ​Alberta Stroke Program Early CT Score.

### Imaging and procedural data

Table 2 provides detailed imaging and procedural data alongside clinical outcomes. Of the patients, 63 (55 ​%) received IVT, while 52 patients (45 ​%) underwent MT alone. The median time from symptom onset to arterial puncture was 210 ​min (IQR, 137–362 ​min). Imaging post-MT was primarily conducted using CT (90 ​%), with MRI used in 10 ​% of cases. The median NIHSS score on day one post-intervention was 7 (IQR, 3–14), and the median FIV was 13 ​mL (IQR, 4–38 ​mL).

**Table 2:** Detailed imaging and procedural data with clinical outcomes.

Variable^a^	N ​= ​147
Intervention, n (%)	
*Both tPA/TNK and thrombectomy*	63 (55)
*Thrombectomy alone*	52 (45)
First line technique, n (%)	
*Aspiration*	33 (24)
*Both*	74 (55)
*Stent retriever*	28 (21)
Side, n (%)	
*Right*	65 (44)
*Left*	82 (56)
Mothership versus drip and ship, n (%)	
*Drip and ship*	26 (30)
*Mothership*	60 (70)
Follow-up infarct volume (ml), median (IQR)	13 (4, 38)
Onset to arterial puncture (min), median (IQR)	210 (137, 362)
Arterial puncture to recanalization time (min), median (IQR)	46 (24, 63)
Onset to recanalization (min), median (IQR)	260 (172, 397)
Onset to IVT needle time (min), median (IQR)	96 (81, 157)
Pre-operative SBP, median (IQR)	150 (134, 170)
Pre-operative DBP, median (IQR)	78 (70, 86)
Pre-operative HR, median (IQR)	75 (68, 87)
Pre-operative glucose (mg/dL), median (IQR)	111 (100, 135)
Anesthesia, n (%)	
*CS/LA*	59 (50)
*GA*	60 (50)
Puncture site, n (%)	
*Femoral*	104 (99)
*Radial*	1 (1.0)
Imaging after MT, n (%)	
*CT*	132 (90)
*MRI*	15 (10)
Total number of passes, median (IQR)	2.00 (1.00, 2.00)
Day one NIHSS, median (IQR)	7 (3, 14)
NIHSS shift, median (IQR)	−3 (−6, 0)
TICI 2c-3, n (%)	62 (42)
TICI 2b-3, n (%)	111 (76)
FPE, n (%)	36 (24)
90-day mRS 0–1, n (%)	50 (34)
90-day mRS 0–2, n (%)	73 (50)
90-day mortality, n (%)	18 (12)
sICH, n (%)	5 (3.6)
ICH (any type), n (%)	31 (35)
HI1, n (%)	1 (1.2)
HI2, n (%)	2 (2.3)
PH1, n (%)	7 (8.1)
PH2, n (%)	3 (3.5)
SAH, n (%)	15 (17)

a Abbreviations: IVT ​= ​Intravenous Thrombolysis, SBP ​= ​Systolic Blood Pressure, DBP ​= ​Diastolic Blood Pressure, HR ​= ​Heart, CS/LA ​= ​Conscious Sedation/Local Anesthesia, GA ​= ​General Anesthesia, sICH ​= ​symptomatic intracerebral hemorrhage, ICH ​= ​intracerebral hemorrhage, TICI ​= ​Thrombolysis in Cerebral Infarction, FPE ​= ​First-Pass Effect, HI1 ​= ​Hemorrhagic Infarction Type 1, HI2 ​= ​Hemorrhagic Infarction Type 2, PH1 ​= ​Parenchymal Hemorrhage Type 1, PH2 ​= ​Parenchymal Hemorrhage Type 2, SAH ​= ​Subarachnoid Hemorrhage.

### Linear regression analysis of infarct volume predictors

The linear regression analysis identified significant factors associated with FIV, as shown in Table 3. Univariable analysis revealed that higher HIR was strongly associated with greater FIV (β ​= ​80; 95 ​% CI, 56 to 103; p ​< ​0.001). Other significant factors included ASPECTS (β ​= ​−10; 95 ​% CI, −14 to −6.3; p ​< ​0.001) and baseline NIHSS (β ​= ​2.0; 95 ​% CI, 0.95 to 3.0; p ​< ​0.001) (Table-3).

**Table 3:** Univariable and multivariable linear regression models of FLAIR Volume Predictors.

Variable^a^	Univariable linear regression Model		Multivariable linear regression Model	
	Β ​= ​(95 ​% CI)^b^	p-value	Β ​= ​(95 ​% CI)^b^	p-value
Male, n (%)	8.4 (−4.6 to 21)	0.2		
Age, median (IQR)	−0.14 (−0.64 to 0.35)	0.57	−0.14 (−0.51 to 0.24)	0.47
Hypercholesterolemia, n (%)	−6.1 (−20 to 7.5)	0.38		
Hypertension, n (%)	5.4 (−10 to 21)	0.49	1.2 (−11 to 14)	0.85
Site of initial occlusion	147			
M2	–			
M3	−14 (−36 to 7.8)	0.21		
A2	14 (−31 to 60)	0.54		
Diabetes, n (%)	−0.63 (−18 to 16)	0.94		
Atrial fibrillation, n (%)	−15 (−31 to 0.66)	0.060	−12 (−24 to 0.30)	0.056
Current smokers, n (%)	−9.4 (−25 to 6.5)	0.24		
Previous use of antiplatelet drugs, n (%)	7.3 (−6.3 to 21)	0.29		
Previous use of anticoagulant drugs, n (%)	−14 (−26 to −1.3)	0.030		
Pre-stroke mRS, n (%)				
*0*	–		–	
*1*	15 (3.4–27)	0.012	9.4 (−1.7 to 21)	0.095
*2*	6.6 (−11 to 25)	0.47	−0.40 (−17 to 16)	0.96
*3*	25 (0.47–50)	0.046	5.1 (−22 to 32)	0.71
ASPECTS, median (IQR)	−10 (−14 to −6.3)	<0.001	−4.0 (−8.0 to 0.07)	0.054
Baseline NIHSS, median (IQR)	2.0 (0.95–3.0)	<0.001	0.52 (−0.30 to 1.3)	0.21
Given IVT, n (%)	5.8 (−7.2 to 19)	0.38		
Stroke cause, n (%)				
*Large artery atherosclerosis*	–			
*Cardioembolic*	−10 (−32 to 12)	0.35		
*Unknown etiology despite work-up*	7.8 (−24 to 40)	0.63		
Intervention, n (%)				
*Both tPA/TNK and thrombectomy*	–			
*Thrombectomy alone*	−2.5 (−18 to 13)	0.76		
Hypoperfusion intensity ratio (HIR), median (IQR)	80 (56–103)	<0.001	40 (21–59)	<0.001
First line technique, n (%)				
*Aspiration*	–			
*Both*	−3.2 (−20 to 14)	0.71		
*Stent retriever*	4.6 (−16 to 25)	0.66		
Baseline imaging, n (%)				
*CT*	–			
*MR*	−29 (−75 to 17)	0.21		
Side, n (%)				
*Right*	–			
*Left*	−5.4 (−18 to 7.6)	0.41		
Mothership versus drip and ship, n (%)				
*Drip and ship*	–			
*Mothership*	0.92 (−11 to 12)	0.87		
Onset to arterial puncture (min), median (IQR)	0.01 (−0.01 to 0.03)	0.51		
Arterial puncture to recanalization time (min), median (IQR)	0.09 (−0.13 to 0.31)	0.43		
Onset to recanalization (min), median (IQR)	0.01 (−0.01 to 0.03)	0.46		
Onset to IVT needle time (min), median (IQR)	0.02 (−0.03 to 0.07)	0.4		
Pre-operative SBP, median (IQR)	0.26 (0.02–0.50)	0.032	0.15 (−0.06 to 0.35)	0.16
Pre-operative DBP, median (IQR)	0.09 (−0.31 to 0.50)	0.65		
Pre-operative HR, median (IQR)	0.07 (−0.30 to 0.43)	0.71		
Pre-operative glucose (mg/dL), median (IQR)	0.23 (−0.06 to 0.52)	0.12		
Anesthesia, n (%)				
*CS/LA*	–			
*GA*	−9.3 (−25 to 6.0)	0.23		
Puncture site, n (%)				
*Femoral*	–			
*Radial*	−31 (−117 to 55)	0.48		
Imaging after MT, n (%)				
*CT*	–			
*MRI*	−10 (−31 to 11)	0.35		
Total number of passes, median (IQR)	5.7 (0.73–11)	0.025		
TICI 2b-3, n (%)	−22 (−36 to −7.2)	0.004	−5.3 (−18 to 7.5)	0.41
FPE, n (%)	−5.6 (−21 to 9.4)	0.46		
sICH, n (%)	11 (−24 to 46)	0.54		
ICH (any type), n (%)	5.8 (−5.4 to 17)	0.31		
HI1, n (%)	11 (−38 to 61)	0.65		
HI2, n (%)	12 (−23 to 47)	0.51		
PH1, n (%)	8.1 (−11 to 27)	0.41		
PH2, n (%)	33 (5.1–61)	0.021		
SAH, n (%)	−8.2 (−22 to 5.7)	0.24		
Embolization in new territories, n (%)	11 (−49 to 71)	0.71		
Perforation, n (%)	5.1 (−33 to 43)	0.79		
Artery dissection, n (%)	10 (−28 to 48)	0.6		

a Abbreviations: mRS ​= ​Modified Rankin Scale, NIHSS ​= ​National Institutes of Health Stroke Scale, IVT ​= ​Intravenous Thrombolysis, SBP ​= ​Systolic Blood Pressure, DBP ​= ​Diastolic Blood Pressure, HR ​= ​Heart Rate, ASPECTS ​= ​Alberta Stroke Program Early CT Score, CS/LA ​= ​Conscious Sedation/Local Anesthesia, GA ​= ​General Anesthesia, sICH ​= ​symptomatic intracerebral hemorrhage, ICH ​= ​intracerebral hemorrhage, TICI ​= ​Thrombolysis in Cerebral Infarction, FPE ​= ​First-Pass Effect, HI1 ​= ​Hemorrhagic Infarction Type 1, HI2 ​= ​Hemorrhagic Infarction Type 2, PH1 ​= ​Parenchymal Hemorrhage Type 1, PH2 ​= ​Parenchymal Hemorrhage Type 2, SAH ​= ​Subarachnoid Hemorrhage.

b CI ​= ​Confidence Interval.

Multivariable analysis confirmed HIR as an independent factor associated with FIV (β ​= ​40; 95 ​% CI, 21 to 59; p ​< ​0.001). Other variables such as ASPECTS (β ​= ​−4.0; 95 ​% CI, −8.0 to 0.07; p ​= ​0.054) and atrial fibrillation (β ​= ​−12; 95 ​% CI, −24 to 0.30; p ​= ​0.056) approached statistical significance.

### Subgroup analysis by occlusion site

The interaction term between HIR and occlusion site was not statistically significant (p ​= ​0.16), indicating no statistically demonstrable effect modification by vascular territory (Fig. 2).

### Correlation between Tmax volumes and follow-up infarct volume

**Fig. 1:**
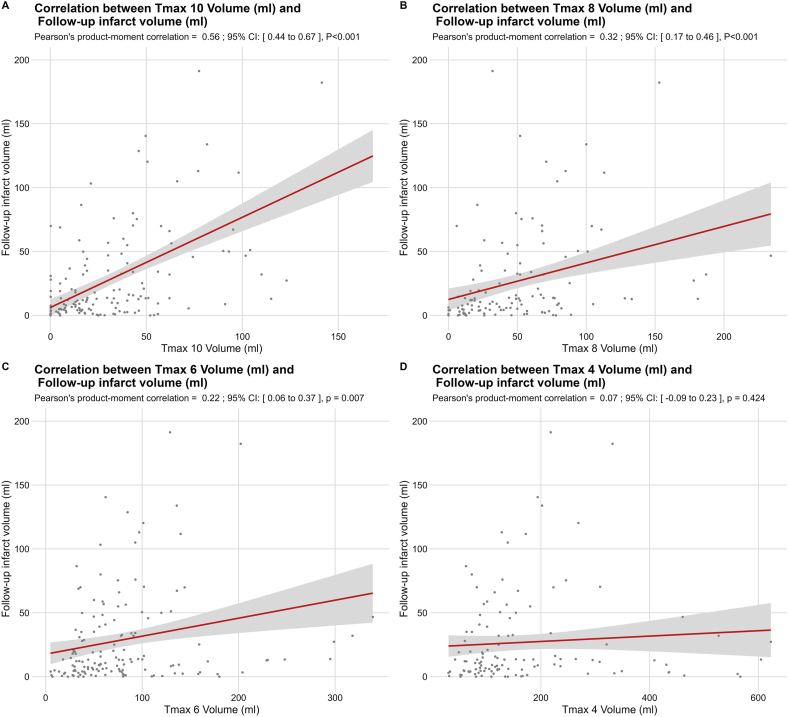
Correlation between Tmax threshold volumes and follow-up infarct volume. Scatter plots show the association of tissue volumes with Tmax >10 ​s (A), >8 ​s (B), >6 ​s (C), and >4 ​s (D) and follow-up infarct volume.

Pearson's correlation analysis demonstrated that Tmax >10 ​s volume had the strongest correlation with FIV (r ​= ​0.56; 95 ​% CI, 0.44–0.67; p ​< ​0.001) (Fig. 1A). Tmax >8 ​s volume showed a moderate correlation (r ​= ​0.32; 95 ​% CI, 0.17–0.46; p ​< ​0.001) (Fig. 1B), while Tmax >6 ​s volume had a weaker but statistically significant correlation (r ​= ​0.22; 95 ​% CI, 0.06–0.37; p ​= ​0.007) (Fig. 1C). There was no significant correlation between Tmax >4 ​s volume and follow-up infarct volume (r ​= ​0.07; 95 ​% CI, −0.09–0.23; p ​= ​0.424) (Fig. 1D).

**Fig. 2:**
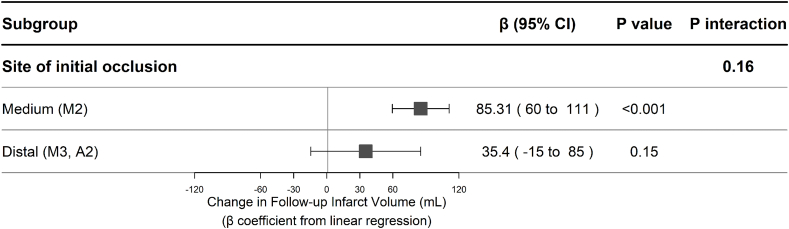
Association between hypoperfusion intensity ratio and follow-up infarct volume stratified by occlusion site.

## Discussion

Our multicenter study demonstrates that a higher HIR, indicative of impaired collateral circulation, is independently associated with larger FIV in patients with MeVO stroke. In multivariable analysis, HIR remained significantly associated with FIV (β ​= ​40 ​mL; 95 ​% CI: 21, 59; *p* ​< ​0.001), even after adjustment for baseline ASPECTS and NIHSS scores.

Notably, our correlation analysis suggests that this relationship is primarily driven by the volume of severely hypoperfused tissue, as defined by Tmax >10 ​s. The Tmax >10 ​s volume showed the strongest correlation with FIV (r ​= ​0.56; 95 ​% CI, 0.44–0.67; p ​< ​0.001), whereas Tmax thresholds representing less severe hypoperfusion (Tmax >6 and ​> ​4 ​s) demonstrated weaker or non-significant correlations. This finding aligns with the study by Yedavalli et al., which found that, particularly in unsuccessfully recanalized patients, Tmax >10 ​s was the optimal threshold for predicting FIV, with superior diagnostic performance compared to other Tmax values [46].

The independent association of HIR and FIV highlights the critical role of collateral circulation for MeVO stroke outcomes [10]. In MeVOs, the role of collateral circulation is poorly characterized. Unlike LVOs, where collateral pathways are often discussed in the context of larger, more accessible vessels, MeVOs involve smaller and more distal arterial branches, making the dynamics of collateral flow distinctly different [47]. The first hours following the occlusion are particularly critical in MeVOs, where smaller vessel networks are tasked with maintaining cerebral perfusion. A lack of sufficient collateral flow in these vessels can predispose to larger infarcts, regardless of the duration between stroke onset and treatment. This observation aligns with studies in MeVO and LVO that point to the role of collaterals for stroke outcomes [15,18,10,48,49].

We also observed that a higher baseline ASPECTS, which indicates less early ischemic injury, is associated with smaller FIV [[50], [51], [52]]. However, ASPECTS can indeed reflect reversible ischemia, as it suggests that lower ASPECTS scores, often considered indicative of extensive brain damage, may not always denote permanent injury but could instead represent areas of potentially reversible ischemia [53]. This is particularly noteworthy as it implies that ASPECTS can help in identifying brain regions where edema, rather than irreversible infarction, contributes to the imaging appearance [54].

An intriguing aspect of our findings is the absence of a significant association between IVT and FIV, despite IVT being standard care for this stroke subtype. A possible explanation for this observation is the limited benefit of IVT as an adjunct to MT in MeVO patients [55,56]. Prior observational studies, including Dmytriw A. et al. study, have reported no clear advantage of IVT in improving outcomes when combined with MT in MeVO strokes [57].

Our study has multiple strengths, including large-scale, multinational, multicenter, and real-world data, thereby improving generalizability. However, our study is not without limitations. First, its retrospective, observational design may be subject to inherent selection bias and unmeasured confounding, despite the use of standardized inclusion criteria across participating centers. Second, the use of CTP as a prerequisite imaging modality may limit the generalizability of our findings, as CTP is more commonly available at tertiary care and comprehensive stroke centers, and less so in community or rural settings [58].

Third, infarct volumes were assessed using both CT and MRI follow-up imaging, and final infarct volumes were adjudicated locally at each site rather than by a central imaging core. While this reflects real-world practice and may enhance external validity, it introduces potential variability due to differences in imaging protocols, segmentation methods, and reader experience. Nevertheless, prior studies have shown high intra- and inter-rater reliability for infarct volume measurements on both CT and MRI, particularly for infarcts larger than 10 ​mL [4,[59], [60], [61], [62]]. Lastly, due to the smaller vascular territory and perfusion volumes in MeVO, HIR measurements may be more prone to technical artifacts, including partial volume effects and segmentation noise, which could affect the precision of Tmax-based perfusion maps and, by extension, the reliability of HIR as a quantitative biomarker in this stroke subpopulation. Future research, ideally involving larger patient cohorts and a broader spectrum of healthcare settings, are essential to validate our findings for stroke treatment and prognosis.

## Conclusion

In this multicenter, multinational study, we demonstrate that a higher HIR is independently associated with larger FIV in patients with MeVO. This relationship is primarily driven by severely hypoperfused tissue (Tmax >10 ​s), highlighting the importance of collateral circulation in these smaller, distal vessels. These results suggest that HIR could serve as a practical imaging marker to guide prognosis and treatment strategies in MeVO strokes.

## Author contribution

**1. Vivek Yedavalli:** Conceptualization, Methodology, Formal Analysis, Writing - Original Draft, Writing - Review & Editing, Visualization.

**2. Hamza Adel Salim:** Conceptualization, Methodology, Formal Analysis, Writing - Original Draft, Writing - Review & Editing, Visualization, Validation.

**3. Dhairya Lakhani**: Investigation, Data Curation, Validation, Writing – Review & Editing.

**4. Basel Musmar**: Resources, Data Curation, Writing – Review & Editing, Project Administration.

**5. Nimer Adeeb**: Resources, Supervision, Validation, Writing – Review & Editing.

**6. Davide Simonato**: Data Curation, Validation, Methodology.

**7. Yan-Lin Li**: Formal Analysis, Data Curation, Visualization.

**8. Orabi Hajjeh**: Data Curation, Resources.

**9. Muhammed Amir Essibayi**: Data Curation, Validation.

**10. Nils Henninger**: Supervision, Writing – Review & Editing, Funding Acquisition.

**11. Sri Hari Sundararajan**: Data Curation, Resources.

**12. Anna Luisa Kühn**: Data Curation, Validation, Writing – Review & Editing.

**13. Jane Khalife**: Data Curation, Investigation.

**14. Sherief Ghozy**: Formal Analysis, Data Curation.

**15. Luca Scarcia**: Data Curation, Resources.

**16. Leonard LL. Yeo**: Supervision, Writing – Review & Editing.

**17. Benjamin YQ Tan**: Data Curation, Validation.

**18. Robert W. Regenhardt**: Supervision, Methodology, Writing – Review & Editing.

**19. Jeremy J. Heit**: Supervision, Writing – Review & Editing.

**20. Aymeric Rouchaud**: Data Curation, Validation.

**21. Jens Fiehler**: Supervision, Resources.

**22. Sunil Sheth**: Writing – Review & Editing, Supervision.

**23. Ajit S. Puri**: Supervision, Resources, Funding Acquisition.

**24. Christian Dyzmann**: Data Curation, Resources.

**25. Marco Colasurdo**: Data Curation, Investigation.

**26. Leonardo Renieri**: Data Curation, Validation.

**27. João Pedro Filipe**: Data Curation, Resources.

**28. Pablo Harker**: Data Curation, Investigation.

**29. Răzvan Alexandru Radu**: Data Curation, Formal Analysis.

**30. Mohamad Abdalkader**: Data Curation, Writing – Review & Editing.

**31. Piers Klein**: Data Curation, Formal Analysis.

**32. Takahiro Ota**: Data Curation, Resources.

**33. Ashkan Mowla**: Data Curation, Validation.

**34. Kareem El Naamani**: Data Curation, Investigation.

**35. Pascal Jabbour**: Supervision, Resources, Funding Acquisition.

**36. Arundhati Biswas**: Data Curation, Validation.

**37. Frédéric Clarençon**: Supervision, Writing – Review & Editing.

**38. James E. Siegler**: Formal Analysis, Writing – Review & Editing.

**39. Thanh N. Nguyen**: Supervision, Project Administration, Funding Acquisition.

**40. Ricardo Varela**: Data Curation, Resources.

**41. Amanda Baker**: Data Curation, Validation.

**42. David Altschul**: Data Curation, Investigation.

**43. Nestor R. Gonzalez**: Supervision, Writing – Review & Editing.

**44. Markus A. Möhlenbruch**: Resources, Supervision.

**45. Vincent Costalat**: Supervision, Resources.

**46. Benjamin Gory**: Supervision, Writing – Review & Editing.

**47. Christian Paul Stracke**: Data Curation, Validation.

**48. Constantin Hecker**: Data Curation, Resources.

**49. Gaultier Marnat**: Supervision, Writing – Review & Editing.

**50. Hamza Shaikh**: Data Curation, Formal Analysis.

**51. Christoph J. Griessenauer**: Supervision, Writing – Review & Editing.

**52. David S. Liebeskind**: Supervision, Methodology, Writing – Review & Editing.

**53. Alessandro Pedicelli**: Data Curation, Resources.

**54. Andrea M. Alexandre**: Data Curation, Validation.

**55. Tobias D. Faizy**: Formal Analysis, Visualization.

**56. Illario Tancredi**: Data Curation, Investigation.

**57. Erwah Kalsoum**: Data Curation, Resources.

**58. Boris Lubicz**: Supervision, Resources.

**59. Aman B. Patel**: Supervision, Writing – Review & Editing.

**60. Maurizio Fuschi**: Data Curation, Formal Analysis.

**61. Max Wintermark**: Supervision, Writing – Review & Editing, Funding Acquisition.

**62. Adrien Guenego**: Methodology, Writing – Review & Editing.

**63. Adam A. Dmytriw**: Conceptualization, Supervision, Methodology, Writing – Original Draft, Writing – Review & Editing, Funding Acquisition.

**All Authors**: Critical revision of the manuscript for intellectual content, final approval of the version to be published.

## Declaration of competing interest

Dr. Regenhardt serves on a DSMB for a trial sponsored by Rapid Medical, serves as site PI for studies sponsored by Penumbra and Microvention, and receives stroke research grant funding from the National Institutes of Health, Society of Vascular and Interventional Neurology, and Heitman Stroke Foundation.

Dr. Guenego reports consultancy for Rapid Medical and Phenox, not directly related to the present work.

Dr. Clarençon reports conflicts of interest with Medtronic, Balt Extrusion (consultant), ClinSearch (core lab), Penumbra, Stryker (payment for reading) and Artedrone (Board); all not directly related to the present work.

Dr. Henninger received support from W81XWH-19-PRARP-RPA form the CDMRP/DoD, NS131756 and U24NS113844 from the NINDS, and NR020231 from the NINR and received compensation from Myrobalan, Inc. and General Dynamics during the conduct of this study unrelated to this work.

Dr. Liebeskind is consultant as Imaging Core Lab to Cerenovus, Genentech, Medtronic, Stryker, Rapid Medical.

Dr. Yeo reports Advisory work for AstraZeneca, Substantial support from NMRC Singapore and is a medical advisor for See-mode, Cortiro and Sunbird Bio, with equity in Ceroflo. All unrelated to the present work.

Dr. Griessenauer reports a proctoring agreement with Medtronic and research funding by Penumbra.

Dr. Marnat reports conflicts of interest with Microvention Europe, Stryker Neurovascular, Balt (consulting), Medtronic, Johnson & Johnson and Phenox (paid lectures), all not directly related to the present work.

Dr. Puri is a consultant for Medtronic Neurovascular, Stryker Neurovascular, Balt, Q'Apel Medical, Cerenovus, Microvention, Imperative Care, Agile, Merit, CereVasc and Arsenal Medical, he received research grants from NIH, Microvention, Cerenovus, Medtronic Neurovascular and Stryker Neurovascular, and holds stocks in InNeuroCo, Agile, Perfuze, Galaxy and NTI.

Dr. Tjoumakaris is a consultant for Medtronic and Microvention (funds paid to institution, not personally).

Dr. Jabbour is a consultant for Medtronic, Microvention and Cerus.

Tobias Faizy was funded by the Else-Kröner-Fresenius-Stiftung (EKFS): Project Number: 2023_EKES.02.

Dr. Siegler has served as a consultant for AstraZeneca, Bayer, and Novartis; has received research funding from the National Institutes of Health (R61NS135583, R01NS114632), Viz.ai, Philips, and Medtronic; compensation from the American Heart Association for editorial services; serves on the Editorial Board for *Neurology* and *Stroke: Vascular and Interventional Neurology*.

Dr.Nguyen reports Associate Editor of Stroke; Advisory board of Brainomix, Aruna Bio; Speaker for Genentech, Kaneka; consulting for Medtronic.
