# Supplementary material for: Hypoperfusion intensity ratio is associated with follow-up infarct volume in medium vessel occlusions: A multicenter multinational study
Source: Neurotherapeutics. 2025 Aug 18;22(6):e00713. doi: 10.1016/j.neurot.2025.e00713 (PMC12664488; doi:10.1016/j.neurot.2025.e00713)
Supplement: Multimedia component 1 [file mmc1.docx]

**Collaborators - MAD MT Investigators**

Abdelaziz Amllay MD^1^, Achala Vagal MD^2^, Adrien ter Schiphorst MD^3^, Ajith J. Thomas MD^4^, Anil Gopinathan^5^, Anne Dusart MD^6^, Carolina Capirossi MD^7^, Charbel Mounayer MD^8^, Charlotte Weyland MD^9^, Cheng-Yang Hsieh MD^10^, Christopher J. Stapleton MD^11^, Erwah Kalsoum MD^12^, Flavio Bellante MD^6^, Géraud Forestier MD^8^, Hamza Shaikh MD^13^, Hugo H. Cuellar-Saenz MD^11^, Iacopo Valente MD^14^, Igor Sibon MD PhD^15^, James D. Rabinov MD^11^, Jérôme Berge MD^16^, Jessica Jesser MD^9^, Juan Carlos Martinez-Gutierrez MD^17^, Kevin Premat MD^18^, Lina Chervak MD^2^, Lukas Meyer MD^19^, Mahmoud Elhorany MD^18^, Miguel Quintero-Consuegra MD^20^, Mohammad Ali Aziz-Sultan MD^21^, Monika Killer-Oberpfalzer MD^22^, Peter T. Kan MD MPH^23^, Priyank Khandelwal MD^24^, Ramanathan Kadirvel PhD^4^, Robert Fahed MD^25^, Sergio Salazar-Marioni MD^17^, Shogo Dofuku MD^26^, Simona Nedelcu MD PhD^27^, Stavropoula I. Tjoumakaris MD^1^, Suzana Saleme MD^8^, Xavier Barreau MD^16^, Yasmin Aziz MD^28^

1 Department of Neurosurgery, Thomas Jefferson University, Philadelphia, PA, USA
2 Department of Neurology and Radiology, University of Cincinnati, USA
3 Department of Neurology, Gui de Chauliac Hospital, Montpellier University Medical Center
4 Departments of Neurological Surgery & Radiology, Mayo Clinic, Rochester, MN, USA
5 Department of Medicine, Yong Loo Lin School of Medicine, National University of Singapore
6 Department of Neurology, Hôpital Civil Marie Curie, Charleroi, Belgium
7 Interventistica Neurovascolare, Ospedale Careggi di Firenze, Florence, Italy
8 University Hospital of Limoges, Neuroradiology Department, Dupuytren, Université de Limoges, XLIM CNRS, UMR 7252
9 Sektion Vaskuläre und Interventionelle Neuroradiologie, Universitätsklinikum Heidelberg, Heidelberg, Germany
10 Neurology Department, Sin-Lau Hospital, Tainan, Taiwan
11 Department of Neurosurgery and Interventional Neuroradiology, Louisiana State University, LA
12 Department of Neuroradiology, Henri Mondor Hospital, Creteil, France
13 Cooper Neurological Institute, Cooper University Hospital, Cooper Medical School of Rowen University, Camden, NJ, USA
14 UOSA Neuroradiologia Interventistica, Fondazione Policlinico Universitario A. Gemelli IRCCS Roma, Italy
15 Neurology Department, Bordeaux University Hospital, Bordeaux, France
16 Interventional Neuroradiology Department, Bordeaux University Hospital, Bordeaux, France
17 Department of Neurology, UTHealth McGovern Medical School, Houston, TX, USA
18 Department of Neuroradiology, Pitié-Salpêtrière Hospital, Paris, France; GRC BioFast. Sorbonne University. Paris VI
19 Department of Diagnostic and Interventional Neuroradiology, University Medical Center Hamburg-Eppendorf, Hamburg, Germany
20 Department of Neurosurgery, Cedars-Sinai Medical Center, Los Angeles, USA
21 Department of Neurosurgery, Brigham and Women's Hospital, Harvard Medical School, Boston MA
22 Departments of Neurology & Neurosurgery, Christian Doppler Clinic, Paracelsus Medical University Salzburg, Austria
23 Department of Neurosurgery, University of Texas Medical Branch, Galveston, Texas, USA
24 Department of Endovascular Neurosurgery and Neuroradiology NJMS, Newark, NJ, USA
25 Department of Medicine, Division of Neurology, The Ottawa Hospital, Ottawa Hospital Research Institute and University of Ottawa, Ottawa, Ontario, Canada
26 Department of Neurosurgery, Tokyo Metropolitan Tama Medical Center, Tokyo, Japan
27 Department of Neurology, University of Massachusetts Chan Medical School, Worcester, MA, USA
28 Department of Neurology, University of Cincinnati Medical Center, Cincinnati, OH
